# Neonatal head and torso vibration exposure during inter-hospital transfer

**DOI:** 10.1177/0954411916680235

**Published:** 2017-01-05

**Authors:** Laurence Blaxter, Mildrid Yeo, Donal McNally, John Crowe, Caroline Henry, Sarah Hill, Neil Mansfield, Andrew Leslie, Don Sharkey

**Affiliations:** 1 Bioengineering Research Group, Faculty of Engineering, The University of Nottingham, Nottingham, UK; 2 Academic Child Health, School of Medicine, University Hospital, The University of Nottingham, Nottingham, UK; 3 Dyson School of Design Engineering, Imperial College London, London, UK; 4 CenTre Neonatal Transport Service, University Hospitals of Leicester NHS Trust, Leicester, UK; 5 CenTre Neonatal Transport Service, Nottingham University Hospitals NHS Trust, Nottingham, UK

**Keywords:** Vibration hazard, shock hazard, linear head acceleration, neonatal, brain injury, monitoring, intraventricular haemorrhage

## Abstract

Inter-hospital transport of premature infants is increasingly common, given the centralisation of neonatal intensive care. However, it is known to be associated with anomalously increased morbidity, most notably brain injury, and with increased mortality from multifactorial causes. Surprisingly, there have been relatively few previous studies investigating the levels of mechanical shock and vibration hazard present during this vehicular transport pathway. Using a custom inertial datalogger, and analysis software, we quantify vibration and linear head acceleration. Mounting multiple inertial sensing units on the forehead and torso of neonatal patients and a preterm manikin, and on the chassis of transport incubators over the duration of inter-site transfers, we find that the resonant frequency of the mattress and harness system currently used to secure neonates inside incubators is ~9Hz. This couples to vehicle chassis vibration, increasing vibration exposure to the neonate. The vibration exposure per journey (A(8) using the ISO 2631 standard) was at least 20% of the action point value of current European Union regulations over all 12 neonatal transports studied, reaching 70% in two cases. Direct injury risk from linear head acceleration (HIC_15_) was negligible. Although the overall hazard was similar, vibration isolation differed substantially between sponge and air mattresses, with a manikin. Using a Global Positioning System datalogger alongside inertial sensors, vibration increased with vehicle speed only above 60 km/h. These preliminary findings suggest there is scope to engineer better systems for transferring sick infants, thus potentially improving their outcomes.

## Introduction

Newborn inter-hospital transfer is important in order to provide the centralised neonatal intensive care delivered in many countries. In the United Kingdom, over 16,000 neonatal transfers occur every year and these are on the increase. Babies are transferred within a trolley mounted incubator, which is heavily laden with equipment. Secure mechanical fixation of this unit gives improved safety in the event of a crash or rapid deceleration and is provided by mounting points in the ambulance floor into which the trolley is then clamped. However, although these precautions mitigate against injury in the event of a road traffic accident, even in the absence of such events, a number of studies have demonstrated an increased rate of mortality and morbidity following neonatal transfer between hospital sites.^1–3^ Of particular concern is the association with brain injury, for example, Levene et al.^4^ found an anomalously high incidence of intraventricular haemorrhage (IVH) in outborn and transferred, compared with inborn neonates. These findings have led to questions regarding other less obvious hazards encountered during transport.^5^ As the incubators, equipment, and staff used during transports are very similar to those in the Neonatal Intensive Care Unit (NICU), and therefore, the care provided remains unchanged, it is suspected that vibration and linear acceleration (i.e. mechanical shock) conditions encountered during transport may be one of the key injury mechanisms. Indeed, a number of animal studies have highlighted the adverse health implications of vibration upon the respiratory system^6^ and cardiovascular status.^7^ In a study of mechanical resonance in the adult brain, Laksari et al.^8^ stated ‘our findings suggest a dangerous frequency, around which relative brain motion is maximized’. Grosek et al.^9^ found ‘an association between daytime road transport and higher heart rate and peripheral blood leukocyte counts, which may be related to some particular (but unidentified) stress of road ambulance transfer’ in a study of 48 transports. Hypothetically, this may in turn result in fluctuating blood flow to brain. Fluctuations in cerebral blood flow are an important mechanism for IVH and subsequent poor neurological outcomes.^10^

In order to be able to mitigate against the deleterious effects of transport, improved knowledge of the damage mechanism would be beneficial. The relative hazard posed by ‘shocks’ (that cause rapid relative motion between skull and brain and hence damage to connective tissue), versus relatively long-term continuous vibration (and its effect upon the body’s control systems, which may produce physiological responses) are unclear.

A number of previous studies have quantified vibration exposure during inter-hospital transport. Shenai et al.^11^ studied vibration during neonatal transports using an abdominal accelerometer. Root-mean-square (RMS) vibration power spectral density was computed, concluding that vibration exposure reaches high levels compared to adult workplace limits. Campbell et al.^12^ measured similar vibration magnitudes, their analysis method differing from Shenai by use of a multi-axis accelerometer, while Gajendragadkar et al.^13^ used manikins together with three-axis accelerometers (on the manikin’s head and transport incubator chassis) to measure both vibration exposure during different transport routes and the isolation properties of mattress configurations. However, these studies did not use standardised methodologies to quantify the hazards associated with whole-body vibration (WBV): Shenai was limited by single-axis measurements, Gajendragadkar failed to validate manikin models against patients, and all three failed to use standardised axis-dependent frequency weightings.

This study aims to provide a baseline assessment of the exposure of neonates to head and torso vibrations, and linear accelerations, that is comparable to both current assessments of vibration in the workplace (ISO 2631-1:1997) and to traumatic head injury in vehicle accidents (HIC_15_). Although extrapolating these adult standards to unwell neonates may be challenging to interpret, it does offer a means of analysis that is well grounded.

A secondary objective is to begin to address the question as to whether transport could be made safer, for example, by a redesign of the incubator or its components, or by exerting routing and speed control over the ambulance’s journey. Therefore, we will also assess the contribution of the mattress type, the road type, and the vehicle speed upon the vibration exposure to the neonate.

## Method

### Design and assembly of a datalogger for micro-electro-mechanical system inertial sensors

As no off-the-shelf device suitable for use in these studies could be found, a datalogger was custom built to record acceleration and angular rotation rate over the course of neonatal transports and routine vehicle transfers between UK hospital sites. This was a two-part device consisting of (1) a base unit containing the power supply (sufficient for 24-h operation) and data storage elements and (2) a set of swappable plugs in cable assemblies (2 m long) incorporating micro-electro-mechanical system (MEMS) sensors.

**Figure 1.**
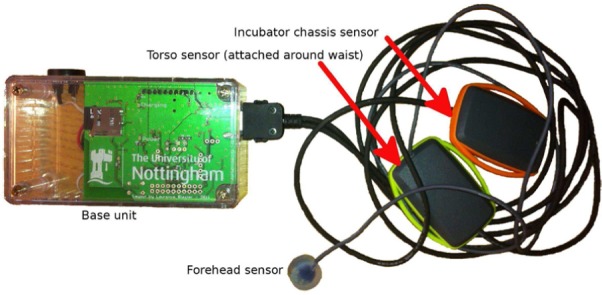
Labelled image of the inertial datalogging system.

These sensor cables incorporated two small (1cm×3cm×2cm) enclosures spaced along their length, with a third smaller sensor board placed at one end, and encapsulated in biocompatible (ISO 10993) silicone resin. Each enclosure contained inertial sensors: a three-axis accelerometer (ADXL345; Analog Devices, Norwood, MA, USA) and a three-axis rate gyroscope with build-in temperature sensor (ITG-3200; InvenSense Inc., San Jose, CA, USA). To reduce the size of the resin-encapsulated sensor for forehead attachment (where a large sensor could potentially induce resonant modes), a combined accelerometer and rate gyroscope integrated circuit (IC) was used (LSM330; STMicroelectronics, Geneva, Switzerland), and this sensor had a 14 mm outer diameter and 3 g encapsulated mass. Figure 1 is a labelled image of the complete system. By incorporating multiple sensor units into a single assembly, aligned datasets could be recorded by a single datalogger, dramatically improving ease of use and reducing the potential for mislabelling in the field.

**Figure 2.**
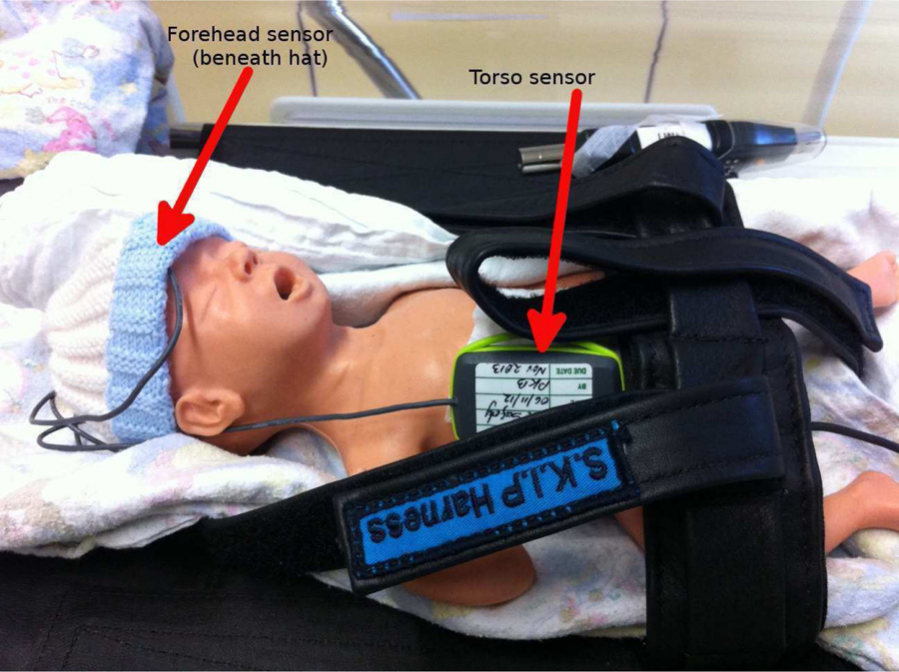
Labelled image of the sensors installed on a neonatal manikin.

Figure 2 is a labelled image of the system installed on the 800 g, 25-week neonatal manikin used in all manikin transports (LifeForm Micro-Preemie; Nasco Inc., Fort Atkinson, WI, USA). The LifeForm Micro-Preemie is designed to mechanically simulate a neonate and was employed in the study for ethical reasons where non-standard procedures may have posed a risk. However, mechanical accuracy in terms of vibration transfer is not claimed by the manufacturer.

A ‘S.K.I.P’ harness (SKIP Harness Ltd, 447A Riddell Road, Glendowie, Auckland, New Zealand), which is routinely used as a patient safety restraint, is seen in this image.

Manikin and neonatal transports used identical incubator (Draeger Air Shields TI500), and sensor configurations, with one sensor enclosure being attached to the incubator chassis (referred to as the chassis site), one to the torso in contact with the ribcage, and finally the silicone resin–encapsulated sensor being secured at the centre of the forehead.

The accelerometers and rate gyroscopes were configured to the largest possible measurement ranges of ±16 *g* and ±2000∘/s, respectively. These were found to be more than adequate for the conditions encountered during transports, with the magnitude of the largest acceleration being 6.5 *g* and largest angular rate being 800∘/s. It was not possible to run all the sensors at their maximum sampling rates due to hardware limitations, so the head-mounted sensor was prioritised, with a high sensor bandwidth of 800 Hz used for the LSM330 accelerometer (Table 1). It should be noted that this is still lower than the 1 kHz specified in the $\mathrm{HI}C_{15}$ standard, meaning that $\mathrm{HI}C_{15}$ impacts may have been slightly underestimated by the sensor.

**Table 1.** Summary of sensor configuration.

Location	Sensor type	Manufacturer	Part no.	Sample rate (Hz)	Anti-aliasing filter (Hz)	Raw bits
Forehead	Accelerometer	STMicroelectronics	LSM330	1600	800	12
	Rate gyro scope and temperature			380	100	16
Torso and chassis	Accelerometer	Analog Devices	ADXL345	100	50	13
	Rate gyro scope and temperature	InvenSense	ITG-3200	100	42	16

### Processing of MEMS sensor data

Analyses of vibration and linear acceleration hazard potential were conducted using the ISO 2631-1:1997^14^ and head impact criterion (HIC)^15,16^ standards, respectively. MEMS accelerometers have significant (∼0.1 *g*) zero-acceleration offsets due to manufacturing tolerances, and their sensitivity typically differs from the published specification by several percent. Offsets typically change with both sensor temperature and device ageing, and so high accuracy calibration can be challenging. For this reason, an accelerometer gain and offset compensation algorithm were used to estimate gain and offset correction terms for each accelerometer sensor axis on a per journey basis. The simple six-point accelerometer calibration process described by Titterton and Weston^17^ was trialled for sensor calibration, but required each sensor to be held stationary for several seconds in six different orientations, a complex procedure that was found to be unreliable when attempted in the field. For this reason a ‘calibration free’ approach was taken, requiring no explicit calibration manoeuvres at any point. The built in *fminsearch* function from the GNU Octave environment was used to optimise a six-component vector (consisting of three offset terms and three gain terms) so as to minimise a cost function defined as the median difference between the lengths of the corrected acceleration vectors and 1 *g*. The procedure converges on the correct gain and offset values under the condition that the median true acceleration is of magnitude 1 *g* and performed reliably in real world application to the recorded datasets.

#### ISO 2631-1 vibration analysis

For the application of ISO 2631 vibration weighting, the horizontal plane frequency-weighting function, $W_{d}$, and vertical plane function, $W_{k}$, were used with torso and forehead data. The motion sickness function, $W_{f}$, was not considered as its appropriateness was unclear, and head weighting used $W_{k}$ as opposed to the $W_{j}$ head vibration function, as there was a soft pillow beneath the head in all transports. Weightings were applied using scripts running in the GNU Octave environment, with filter coefficients generated using the procedures outlined by Rimell and Mansfield.^18^

As ISO 2631 defines sensor axes relative to a standard body position, movement of the patient, and practical considerations when mounting the transducers on the patient created a significant challenge. This problem was overcome using a signal processing algorithm (similar to those used in auto-pilot systems) to track orientation and transform the acceleration data into vertical and horizontal plane components that correspond with ISO 2631 weighting schemes. The three-axis sensor data from the accelerometer and rate gyroscope on each sensor were transformed from ‘sensor body’ (*x*, *y*, *z*) or local sensor axis co-ordinates to a fixed ‘world’ co-ordinate system (N,E,D) of fixed horizontal (North, East) and vertical (Down) axes.

**Figure 3.**
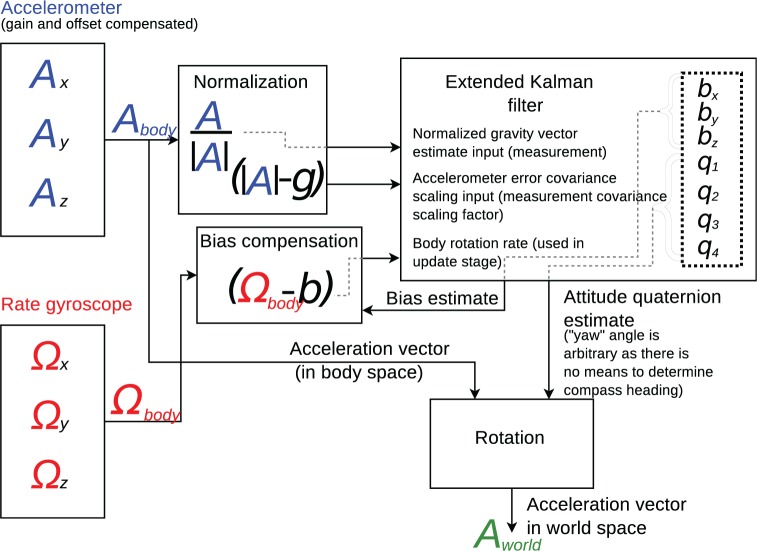
Flow diagram showing data flow through the orientation tracker.

The orientation tracking algorithm is illustrated schematically in Figure 3. An extended Kalman filter (EKF) was used, with a seven-component state vector consisting of three gyroscope bias terms ($b_{x,y,z}$, the estimated rate gyroscope output in a stationary state), and estimated attitude quaternion (sensor body relative to world co-ordinate system). In most previous strap-down inertial measurement unit (IMU) systems, a magnetometer has been used to allow orientation around the vertical axis (yaw) to be determined. No magnetometer was used here, as absolute heading was not required for sensor re-orientation.

The orientation tracker is based on the algorithm described by Foxlin^19^ (Figure 2, the ‘Direct Kalman’ approach), with modifications: no magnetometer; the accelerometer measurement error covariance matrix scaled using the absolute difference between the magnitude of the measured acceleration and a 1 *g* acceleration (i.e. $9.8m/s^{2}$). This covariance scaling reduced the influence of prolonged vehicle acceleration on gyroscope bias and orientation; a prolonged period of acceleration leads to a non-vertical acceleration vector (biassing the filter), but also to an acceleration with magnitude greater than 1 *g*, allowing such acceleration events to be identified and assigned a high error when input into the EKF.

Once the acceleration vectors had been rotated into the NED frame ($A_{world}$ in the figure), it was assumed that the patient was in a reclining position, and that the ambulance chassis was horizontal. Although neither of these conditions will be completely satisfied in practice, it was judged that any deviations of the vehicle from horizontal were likely to be of short duration and limited tilt angle (likely <5∘), making them a less significant cause of mis-modelling than imperfect patient orientation relative to a true reclined position, an error source that cannot easily be controlled. It should also be noted that the ISO 2631 specification states that ‘the sensitive axes of transducers may deviate from the preferred axes by up to 15∘ where necessary’. In the world or NED frame, North was treated as patient’s *z*-axis, East as patient’s *y*-axis, and Down as the patient’s *x*-axis. The ISO 2631 weighting filters could then be applied (using the algorithm described by Rimell and Mansfield^18^) giving weighted outputs at the raw accelerometer sample rates.

Further windowing of weighted data into 10-s intervals, followed by calculation of the RMS vibration on a per window basis, was then undertaken, allowing analysis of vibration as a function of road class and vehicle speed, with the 10s period being chosen based upon consideration of typical vehicle acceleration. Calculation of crest factor and 8h equivalent vibration exposure, or A(8) proceeded as per ISO 2631 (7.2.3).

#### HIC_15_ analysis of linear head acceleration

The HIC is defined by the National Highway Traffic Safety Administration (NHTSA),^16^ chapter 2. HIC is the acceleration in *g* units integrated with respect to time over a rolling window and raised to the power of 2.5 following normalisation with respect to the integration period (equation (1)). HIC can be defined over arbitrary window lengths, with these usually being denoted in ms using a subscript. $\mathrm{HI}C_{15}$ was implemented as 15 ms as this is a common window length, used in, for example, the European Enhanced Vehicle-Safety Committee (EEVC) working group report^20^

(1) $$\mathrm{HIC}=(t_{2}-t_{1}){\left[\frac{\overset{t_{2}}{\underset{t_{1}}{\int }}a(t)\mathrm{dt}}{t_{2}-t_{1}}\right]}^{2.5}$$

$\mathrm{HI}C_{15}$ was assessed using the head sensor accelerometer. A rolling window method was used to process the $A_{world}$ accelerometer data (which was gain and offset compensated and rotated into world space, but not changed in magnitude by the quaternion rotation), with local maxima of HIC^15^ above a threshold of 0.3 being logged.

Although extrapolation of $\mathrm{HI}C_{15}$ thresholds down to neonatal patients is difficult, extrapolation to newborn and 12-month-old subjects has been attempted, for example, Eppinger et al.^16^ suggested a limit value of 390 for 12-month-old subjects and noted that values as low as 200 have been suggested elsewhere. Using the formula $\lambda_{HIC}=\lambda_{\sigma f}^{2.5}-\lambda_{L}^{-1.5}$, where $\lambda_{HIC}$ is the scaling factor for HIC, $\lambda_{\sigma f}$ is for tissue mechanical maturity, and $\lambda_{L}$ is for length, Johannsen et al.^21^ discuss scaling of HIC with age (from the validated Q3 dummy and with data from the CASPER project). In this study, using tensile strength data of a 22-week foetus and the dimensions of the manikin gives a scaling factor of $\lambda_{HIC}=0.13$. Applying this scaling factor to those validated for the Q3 dummy gives injury thresholds of $\mathrm{HI}C_{15}=99$ and 130 for 20% and 50% risks of an Abbreviated Injury Scale (AIS) 3+ injury, respectively. The EEVC working group 12 and 18 report^20^ reaches very similar figures using the CHILD project dataset.

#### Analysis of vehicle speed and road class

An off-the-shelf Global Positioning System (GPS) datalogger (M-1200E; Holux Technology Ltd, Taiwan), installed in the vehicle cab recorded vehicle position and velocity over the course of all journeys. As data collection took place over several months, a correlation technique (between vibration and GPS vehicle speed) was employed to ensure correct data alignment (to within 5 s).

Road classification used the open access ‘nominatim’ server run by OpenStreetMap (nominatim.openstreetmap.org/reverse) to convert GPS positions to location descriptors, which were then passed back to the server to find the nearest object described as a ‘way object’ (implying a road). As this takes several seconds, optimised querying was employed: the raw GPS data (a 1Hz time-series record) were interpolated to a physically uniform sequence of points, one per 100 m of travel, and binary search was applied to identify transition points between road classes with minimal queries. Retrieved ‘way objects’, passed back from the server as xml files, were finally processed with string matching and classified as one of nine road types: motorway, expressway, trunk, primary, secondary, tertiary, tertiary link, service, and unclassified.

To explore the influence of road class and vehicle speed upon vibration, the 10s RMS-weighted vibration intervals were matched to the corresponding road class and vehicle speed and then binned to calculate the total number of intervals (and thus travel duration) falling within a range of RMS vibrations ($0.1m/s^{2}$ increments), and vehicle speeds (10 km/h increments) for the entire dataset.

### Selection of patient and manikin transports

A convenience sample was recruited, consisting of infants requiring transfer by the CenTre transport service between Trent Perinatal Network neonatal units. Patient inclusion criteria were pragmatic, including all infants who were cared for in a neonatal unit and required transfer, but excluding those undergoing palliative care. Manikin studies took place on ambulance journeys when no patient being transferred (i.e. before or after transferring a patient), allowing these journeys to serve as direct validators: using the same transport system, vehicle, and routes. The CenTre service routinely uses a sponge mattress for transfer and a ‘S.K.I.P.’ harness as the patient safety restraint.

To investigate the influence of mattress type upon vibration, the manikin studies were divided into three groups, which used: the stock Draeger incubator mattresses, a polyurethane foam material (sponge) also used in all neonatal patient transports; gel mattresses (Squishon; Philips Healthcare); and air mattresses (Repose Babynest, Frontier Therapeutics, Blackwood, UK).

The journeys studied were within a group of 16 hospital sites, and all but four of the routes were unique. The UK transport fleet consists of numerous vehicle types, but to minimised confounding effects, transports were constrained to a single vehicle make and model: Volkswagen LT-35 (3 L diesel). The LT-35 has dual wishbone front suspension with a leaf-sprung rear live axle, a layout used in most of the UK vehicle fleet, and similar to the Chevrolet P-30 employed in a previous study by Shenai et al.^11^ Informed consent was obtained in writing from the patient’s parents or legal guardian before all neonatal patient transports commenced.

## Results

Data were recorded from a total of 35 ambulance journeys, 12 of which were neonatal transports, with the others using the manikin. Total duration was 34 h, with journey distances being between 45 and 200 km. The 12 neonatal transports involved a total of 10 patients, birth gestation range 24+2–41+2 weeks, and weight range 0.9–4.6 kg, median 1.9 kg.

The forehead sensor data from three manikin journeys were found to be corrupted due to intermittent faults with the sensor cabling, leaving 32 journeys with data from all three sensor sites. Out of the manikin transports, 10 (including 8 with forehead sensor data) used a sponge mattress, 7 (6 with forehead sensor data) a gel mattress, and 6 an air mattress.

### Frequency space vibration analysis

**Figure 4.**
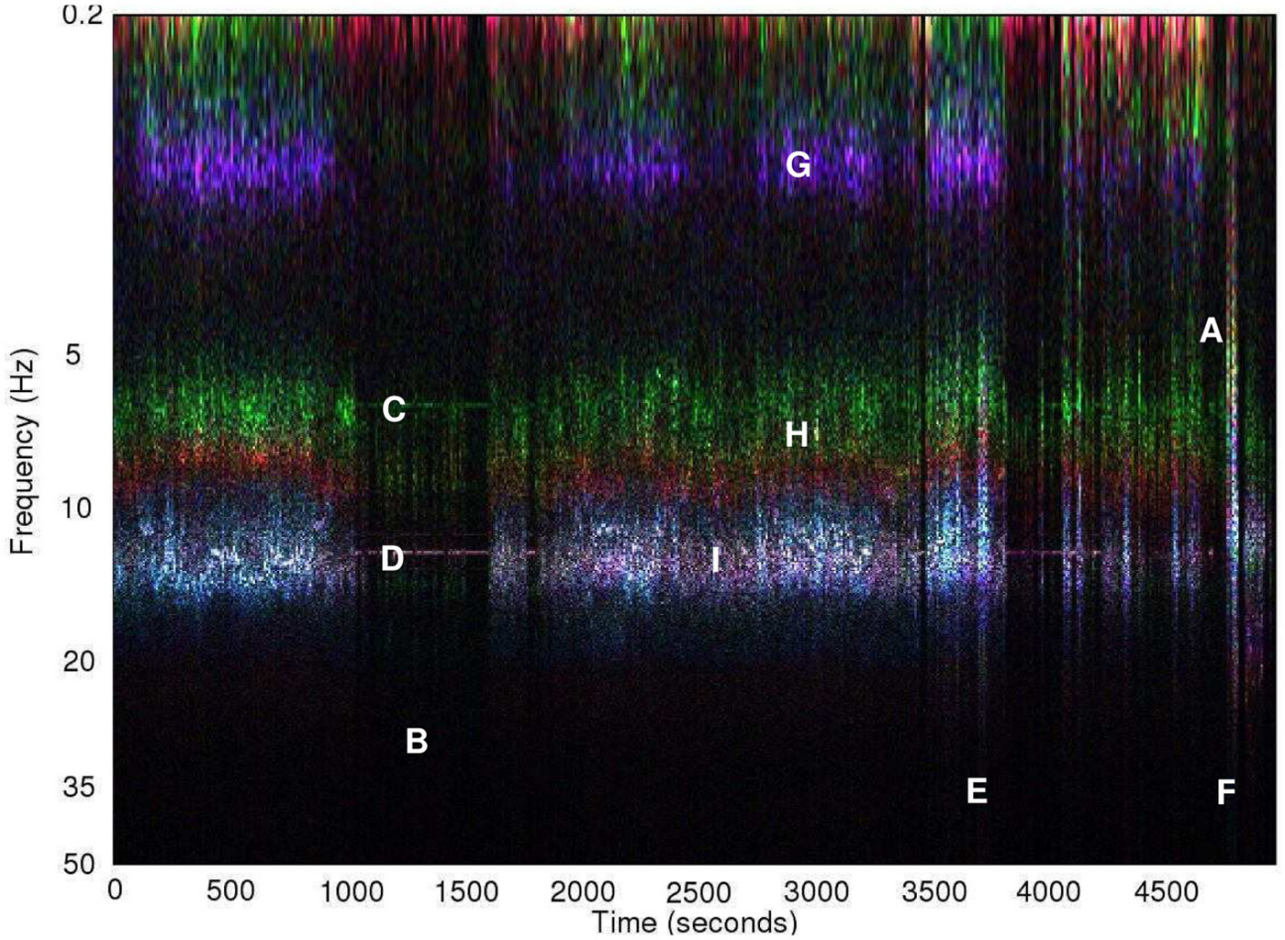
Spectrogram of raw acceleration during a typical transport, forehead site.

Figure 4 shows a spectrogram (using 10s bins) of vibration at the forehead site over the course of a typical manikin transport with sponge mattress. The *x*, *y*, and *z* sensor axes are mapped to red, green, and blue, respectively, in the image, meaning that the colour separation indicates vibration along different axes.

Several features correspond to events noted during the ambulance journey: (1) periods of very low vibration when the vehicle was stationary (vertical bands, for example, at time A), with the engine idling during some of these (e.g. time B), this leading to horizontal line C at approximately 7 Hz with 14 Hz harmonic D and (2) short periods of broad spectrum noise. There are two short periods of broad noise, at times E and F, which correspond to times when the manikin and datalogging equipment was repositioned, leading to short mechanical shocks. The vibration occurs along two distinct bands; G at 1.5 Hz and a band spanning from 6.5 Hz (H) to 13 Hz (I). This second band has clear colour separation between H and I, indicating that the vibration axis is frequency dependent and thus this band may be the result of multiple resonances. Most vibration energy was concentrated between 5 and 20 Hz. The fact that colour of the engine vibration harmonics during idling matches that of the bands at approximately 7 and 14 Hz (when the vehicle is in motion) suggests that these bands may originate from the engine, as the vibration direction is similar. The lower frequency band G appears to arise from resonance of the vehicle suspension.

**Figure 5.**
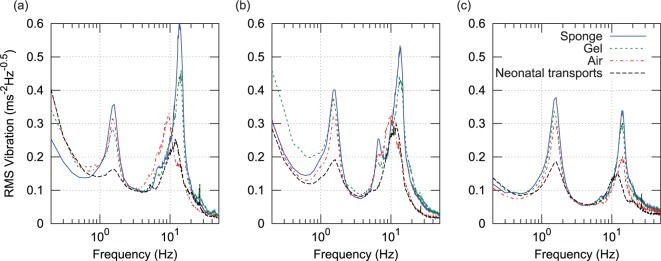
Spectrum of vertical (*z*) acceleration magnitude, all three sites and four journey types: (a) head, (b) torso, and (c) incubator chassis.

**Figure 6.**
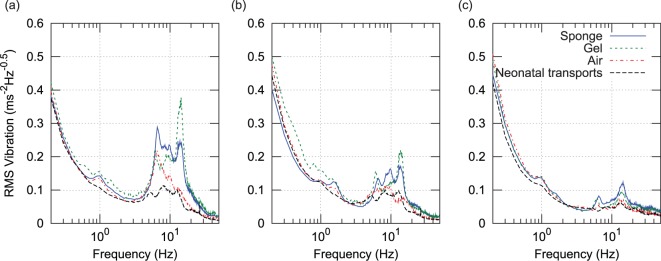
Spectrum of horizontal plane (*x*, *y*) acceleration magnitude, all three sites and four journey types: (a) head, (b) torso, and (c) incubator chassis.

In Figures 5 and 6, vibration spectral density has been plotted following subdivision of the data according to sensor site, axis (horizontal plane or x/y, and vertical or *y*-axis), and journey type. Journeys were divided into four types; three manikin types according to mattress configuration (sponge, gel, or air), and neonatal transports as a fourth group (these used the sponge mattress). For each sensor site and each axis, RMS vibration spectral density was calculated by averaging the appropriate data from all of the journeys of each of the four journey types. These types are indicated according to the figure key. Two very distinct vibration peaks can be seen in the figures. The lower frequency peak aligns closely with the band identified earlier in the spectrogram as G and likely originating from resonance of the vehicle suspension. The higher frequency peak spans the 7 to 14 Hz region which was earlier identified as likely originating from the engine and transmission. The small spike at 26 Hz in the neonatal transports was found to have originated from an incubator ventilation fan which was turned off during manikin transports. The vertical axis acceleration magnitude spectrum at the torso site recorded during neonatal transport (Figure 5(b)) shows very good agreement to the results published by Shenai et al.^11^ (Figure 1) who used a piezoelectric accelerometer with a 2–30 Hz bandwidth at the same sensor site.

Compared to the incubator chassis, spectral density in the 3–20 Hz range is up to four times higher at the torso and forehead sensor sites, indicating that vibration is amplified over this spectral range. This finding is in agreement with a previous study by Gajendragadkar et al.,^13^ in which the mattress system amplified vibration magnitude by factors of between 2.2 to 3.4 (sponge) and 1.1 to 2.1 (gel type). However, this previous study is not directly comparable, as it compared RMS values computed from 50 Hz low-pass-filtered acceleration data.

To analyse exposure hazard, a model was used consisting of an input ‘chassis level’ function (the RMS vibration spectral density at the chassis site), passed through a frequency-dependent weighting function ($W_{k}$ or $W_{d}$ as per ISO 2631), and finally through a site- and configuration-dependent gain function (computed from the study data) modelling resonant effects between the incubator chassis and the neonate or manikin. Overall, RMS vibration at the head and torso sites could then be calculated for the various mattress configurations. Compared to a direct comparison of weighted RMS vibration between study groups, this method reduces confounding effects from journey to journey variance in chassis vibration, allows for insight into the origin of the vibration hazard, and enables evaluation of configurations that were not part of the original dataset. It may thus be possible to extend the analysis to configurations such as neonate with air mattress, although this was not attempted as differences between the manikin and neonates were minimal.

Figure 5(c) shows a 45% reduction in the amplitude of the 1.5-Hz peak during neonatal transport. It would appear that drivers may have been more attentive to road conditions and vehicle vibration during patient transport, a hypothesis supported by the reduction in both frequency and amplitude of the higher frequency (engine vibration related) peak. For this reason, two ‘chassis level’ spectral density functions were generated, one from manikin transports and the other from neonatal transports.

**Figure 7.**
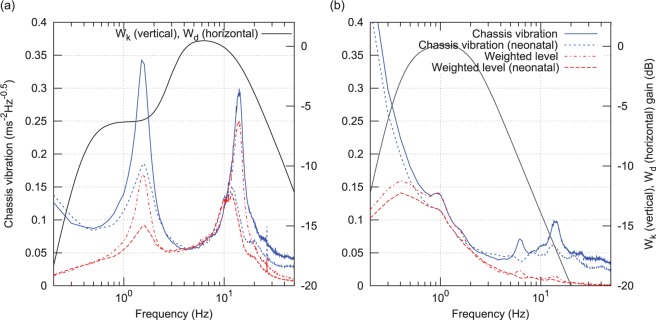
RMS chassis vibration in vertical axis and horizontal plane, before and after application of ISO 2631 weighting functions $W_{k}$ and $W_{d}$: (a) vertical axis and (b) horizontal plane.

Application of the frequency-weighting functions $W_{k}$ and $W_{d}$ to the chassis vibration is illustrated in Figure 7 as the ‘Weighted level’. The peak in vertical axis weighted vibration spectral density at around 12 Hz can be seen to dominate this damage potential function. Mattress configurations, driving styles, or vibration damping arrangements, which minimise the amplitude of this peak, would appear to be a promising route forward if a reduction in overall vibration exposure level is desired.

The vibration gain functions were calculated between the incubator chassis and the forehead and torso sensors by dividing torso and head spectral density functions by the chassis spectral density. This allowed for assessment of both of the effects of different mattress configurations upon vibration attenuation and of the validity of the manikin as a mechanical model through comparison with the neonatal patients.

**Figure 8.**
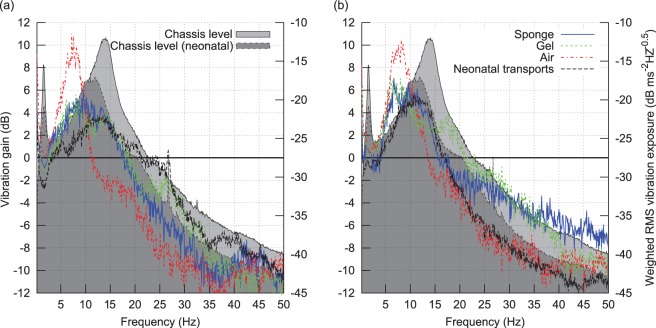
Vertical (*z*) acceleration gain between incubator chassis and neonatal and manikin sensor: (a) head and (b) torso.

Figure 8 plots vibration gain between the incubator chassis and both the manikin (with sponge mattress) and the neonatal patients. Although the two gain functions deviate by up to 3 dB (∼40%) in places, there is a less than 1 dB (∼10%) deviation over the 10–18 Hz frequency band, where gain is highest and the majority of the damaging vibration is concentrated (Figure 5(c)). The spike around 26 Hz in the neonatal trace is caused by the raw data peak identified earlier (and originates from a fan). Given the close match between gains, it appears that the manikin is an appropriate model for the assessment of neonatal vibration exposure. The close match is also notable, given the large range of patient masses across the study group (0.9–4.6 kg), which did not overlap with the 0.8 kg manikin mass. This indicates that patient mass only weakly affects vibration damping, and that the heterogeneity of the sample group is unlikely to have a significant impact on the results.

**Figure 9.**
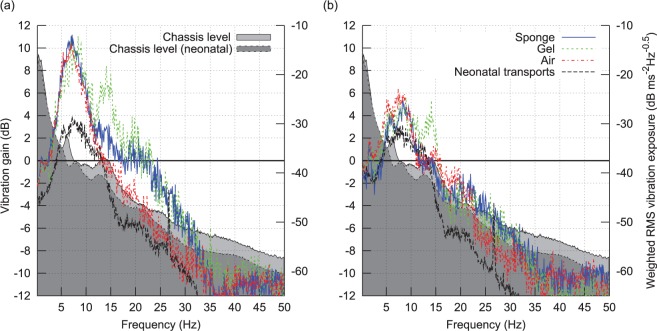
Vertical (*z*) acceleration gain between incubator chassis and (a) head, (b) torso. Plotted versus frequency for all four journey types.

**Figure 10.**
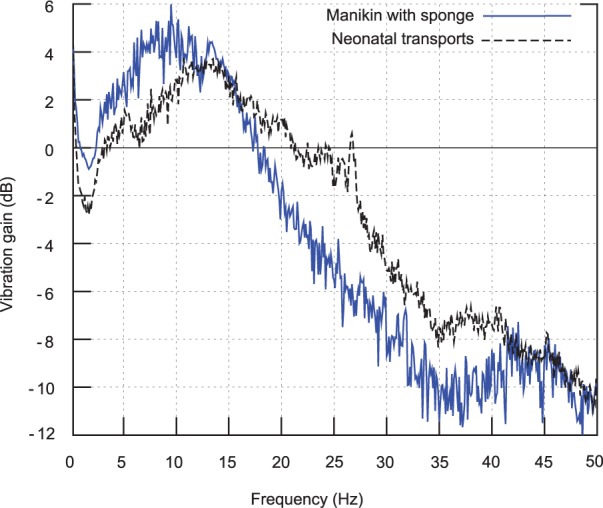
Horizontal plane (*x*, *y*) acceleration gain between incubator chassis and (a) head, (b) torso. Plotted versus frequency for all four journey types.

Vibration gain (in decibels) was also calculated between the incubator chassis and the forehead and torso sensors for the four different journey types and is plotted in Figures 9 and 10. The plot lines (left-hand side axes) are vibration gains in decibels, and the dB scaled chassis sensor vibration spectral density, or ‘Chassis level’, is plotted as a shaded background (right-hand side axes). A gain peak (indicating resonance) is seen at 7 Hz for the air mattress and around 10 Hz for the other mattress configurations, with a peak gain of between 3 and 5 dB in the vertical axis for both the sponge and gel mattresses, and the neonatal transports, with a considerably higher peak for the air mattress in the vertical axis (10 dB for the head at 7 Hz). The response rolls off at higher frequencies at approximately 15 dB per decade in the vertical axis (i.e. slightly lower than a first-order roll-off) and around 20 dB per decade in the horizontal axis from 5 to 35 Hz (first order).

It is notable that although there is a 7 dB peak at 14 Hz in the horizontal axis for the gel mattress which is not present for the sponge mattress, the vibration gain of the gel mattress is similar to the sponge mattress, with the gel gain being within 2 dB of the sponge over most of the plotted frequency range, thus suggesting that the sponge and gel mattresses are mechanically similar. It is suspected that the large 7 dB deviation in horizontal plane attenuation at the head site around 7 Hz (where the large resonance peak is strongly damped in the neonates) may be related to the different procedures used to secure the neonates as compared to the manikins; since for the neonatal transports, air lines, and so on need to be attached, changing the mechanical coupling to the head.

Another significant feature seen in the two vertical axis gain plots is that peak gain occurs at a lower frequency than at a peak ‘Chassis level’ (i.e. chassis vibration spectral density during manikin transports) for all four transport types. For neonatal transports, the peak vertical vibration density, or ‘Chassis level (neonatal)’, occurs at approximately 12 Hz, and so is lower than the 14 Hz peak of the ‘Chassis level’, and roughly coincidies with the resonance peak of both the sponge and gel mattresses with manikin, and the neonate (with sponge mattress) configurations, meaning incubator chassis vibration will drive the mattress and manikin/neonate system on resonance. However, as the peak in air mattress gain is at a considerably lower frequency of 7 Hz, it avoids such an effect.

Finally, the two frequency-weighted chassis vibration spectral density functions (Figure 7) were scaled using the vibration gain functions (Figures 9 and 10), and the overall RMS vibration at the head and torso sites was calculated for the four mattress configurations (manikin with sponge, gel, and air, neonate with sponge) and two chassis level functions (for manikin and neonate driving styles).

**Figure 11.**
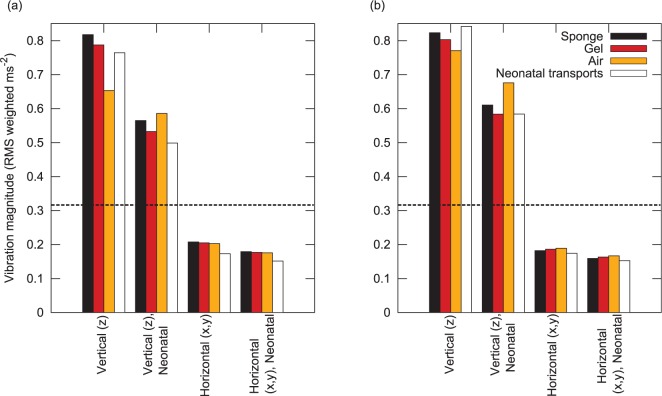
RMS vibration in vertical axis and horizontal plane, after application of ISO 2631 weighting functions and vibration gain to the two chassis vibration density functions. The dashed horizontal black line de-marks the ISO 2631 ‘comfort limit’ of $0.315m/s^{2}$: (a) head and (b) torso.

The resultant RMS vibration is plotted in Figure 11. Several features are seen: the vertical axis vibration dominates overall exposure; the small changes in driving style during the neonatal transports reduced vibration magnitude by approximately 30%; the air mattress results in an increase in vibration exposure with the neonatal driving style; and there is virtually no difference between the manikin with sponge mattress, manikin with gel mattress, and neonate with sponge mattress. Use of an air mattress results in only a modest (∼15%) reduction in vibration with the manikin driving style. RMS vertical axis vibration is well above the $0.315m/s^{2}$ comfort limit specified in ISO 2631, with horizontal plane vibration falling well below this limit.

Thus, as well as illustrating the severity of the vibration experienced during neonatal transport, these preliminary results also suggest that replacement of sponge mattresses with air or gel mattresses may not be beneficial; the gel mattress does not result in any significant reduction in vibration exposure, and the air mattress results in slightly increased vibration exposure when the vehicle is driven within a low-engine speed range. Furthermore, the gel mattress has a considerably higher density (approx 1 g/mL) than the sponge (approximately 0.2 g/mL), presenting an increased risk of injury in the event of a road traffic accident.

### HIC_15_ data

$\mathrm{HI}C_{15}$ values were extremely low across the entire dataset. For this reason, a more detailed analysis of the $\mathrm{HI}C_{15}$ data was not conducted. Out of the 12 transports with neonatal patients, $\mathrm{HI}C_{15}$ exceeded a threshold of one on only four journeys, with a maximum value of 9.3 being seen at the forehead sensor on one journey. The majority of patient transports saw no $\mathrm{HI}C_{15}$ impacts above a threshold of 0.3, and the maximum $\mathrm{HI}C_{15}$ at the forehead sensor site during manikin transports was 13.1. These values are well below both the accepted limit value of 700 for adults,^16^ and the scaled value derived in section ‘$\mathrm{HI}C_{15}$ analysis of linear head acceleration’ of 99 for 20% risk of AIS 3+. Since the maximum $\mathrm{HI}C_{15}$ values for neonatal (9.3) and manikin (13.1) transports were an order of magnitude smaller than these thresholds, it would appear to be highly unlikely that linear acceleration shocks will directly lead to significant traumatic head injury.

### A(8) exposure statistics

The A(8) metric is defined in ISO 2631 and measures cumulative WBV exposure, normalised to an 8-h equivalent period using a nonlinear scaling. WBV is defined as the quadratic mean of the weighted horizontal plane and vertical axis vibration. The European Union (EU) vibration directive defines a daily exposure action value of $0.5m/s^{2}$ for A(8) occupational exposures to WBV.^22^

**Figure 12.**
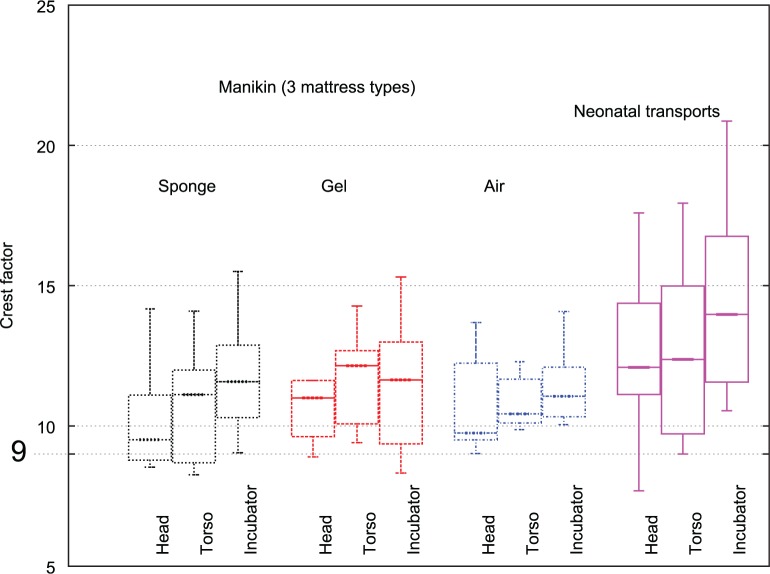
Crest factors (ISO 2631 standard).

ISO 2631 warns that A(8) may prove inaccurate in the case of a high crest factor (the ratio of peak filtered vibration magnitude to RMS vibration over the exposure interval). A fourth power integration method is suggested as more appropriate in cases where crest factor is greater than nine, but fourth power exposure values are more difficult to interpret. Crest factor was calculated on a per journey basis and was greater than nine for the majority of transports (Figure 12).

**Figure 13.**
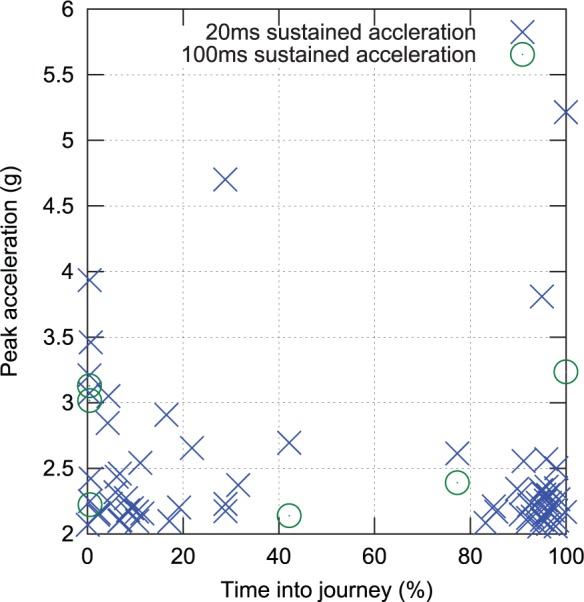
Shock events (defined as a greater than 2 *g* acceleration sustained for more than 20 or 100 ms) over the course of all transports, time normalised to journey duration.

As it was hypothesised that crest factors may be biassed by a small number of acceleration events during vehicle loading and unloading, the raw acceleration data were processed to locate instances of high acceleration. This analysis is plotted in Figure 13 and supports the hypothesis, with shock events clustering around the start and end of journeys. Note that sensors were secured to the manikin or neonate prior to the datalogger being turned on, and before the transport trolley was loaded into the transport vehicle.

This process was repeated in reverse order once the vehicle reached its destination, with the datalogger turned off prior to sensor removal. Thus, the captured events are believed to represent real accelerations, rather than simply attachment and removal of the sensors. Exclusion of 5% of the data at the beginning and end of each journey to avoid inclusion of these clusters lowered median head crest factor to 7.8 and torso to 8.9. For these reasons, it would appear that A(8) is an appropriate means of assessing cumulative exposure, as median crest factors over the middle 90% of the transport period (when the vehicle was in motion and the patients were exposed to the majority of the hazardous vibration) are below nine.

**Figure 14.**
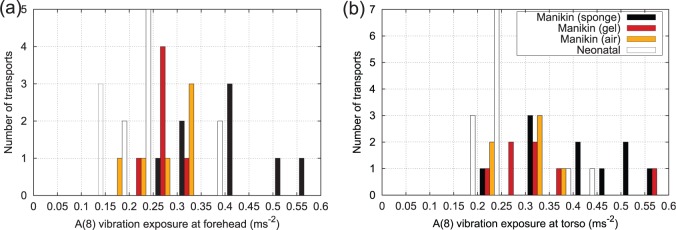
Histogram of A(8) exposure for all journeys: (a) forehead site and (b) torso site.

A(8) exposures were calculated from the weighted acceleration data on a per journey basis. From Figure 14(a) and (b), two journeys saw an A(8) vibration exposure magnitude greater than $0.5m/s^{2}$ at the forehead. These did not include any neonatal transports. Vibration exposure to the torso was slightly worse, with four transports exceeding $0.5m/s^{2}$, although again this included no neonatal transports. Nevertheless, the fact that neonatal exposures approach the workplace exposure limit for healthy adults would appear to be a significant cause for concern.

### Analysis of vibration versus vehicle speed and road class

**Figure 15.**
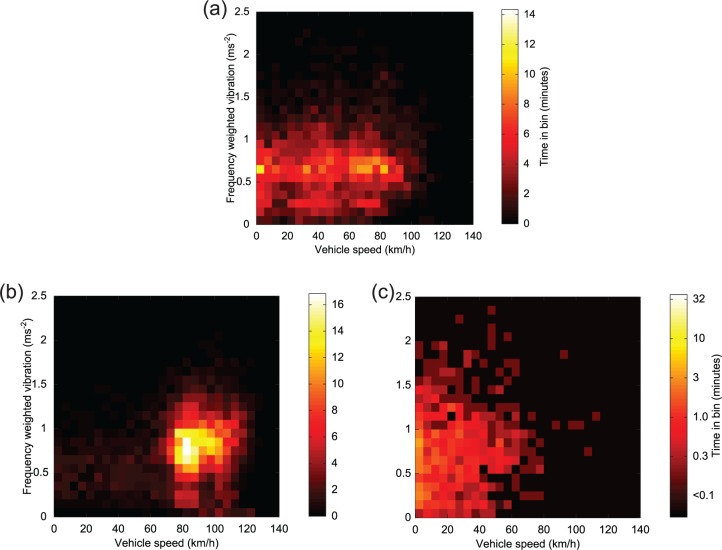
Forehead vibration versus speed: (a) ‘A’ and trunk road classes, (b) motorway and expressway classes, and (c) remaining (lower) road classes (logscale).

Vehicle vibration versus vehicle speed and road class is plotted in Figure 15. The binning procedure used in this analysis was outlined in section ‘Analysis of vehicle speed and road class’. As a relatively large period of time was spent with the vehicle at low speeds on minor roads, a logarithmic colour bar has been used in one of the figures for clarity.

Maximum recorded vehicle speed was 131 km/h, with 99% of travel time spent below 112 km/h (70 mile/h), and a median speed of 75 km/h (47 mile/h). Vehicle speed was strongly related to road class; speeds exceeded 90 km/h over only a very small proportion of ‘A’ road travel time, whereas almost all motorway and expressway class travel was between 80 and 120 km/h, and most minor class road travel was below 50 km/h. This complicates any analysis of the effect of road class upon vibration. At a given speed, vibration might be expected to be inversely related to road grade, but any such effects appear to be small. Comparing Figure 15(a) and (c), lower class roads have a slightly higher median vibration at vehicle speeds between 5 and 50 km/h, suggesting a poorer ride on ‘B’ and lower class roads. Any comparison in ride quality between ‘A’ and expressway/motorway classes (Figure 15(b)) is impossible as there is no significant overlap in speed ranges. From Figure 15(a), the vibration distribution during periods when the speed was 0–10 km/h is very similar to that at speeds below 70 km/h. As the engine was usually idling when the vehicle was stationary (Figure 4), this suggests that engine vibration may be the most significant vibration source at these speeds.

**Figure 16.**
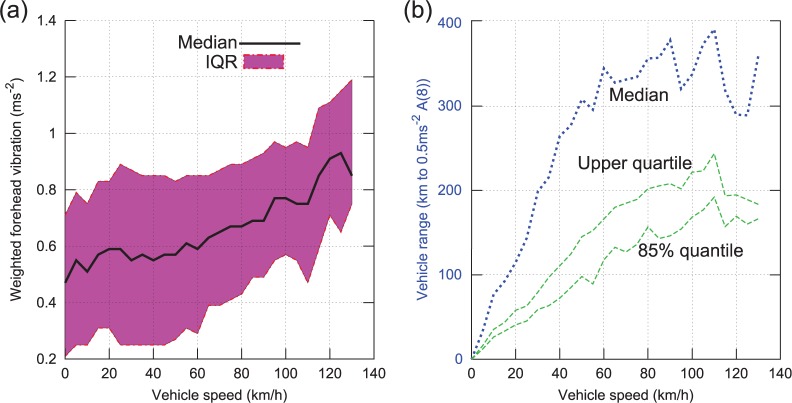
(a) Median vibration and quartiles versus vehicle speed and (b) vehicle range before A(8) exposure limit exceeded versus vehicle speed.

Although it complicates analysis of the influence of road type upon vibration, the very strong dependence of speed upon road type leads to important conclusions regarding choice of transport route; for example, if a speed above 85 km/h is desirable, then a route involving expressway or motorway class roads should be chosen, as lower speeds are found on ‘A’ classes. To further investigate the relationship between vehicle speed and exposure, the median and quartiles of the weighted forehead vibration were calculated as a function of vehicle speed (using all data recorded over all road classes) and are plotted in Figure 16 (left subplot).

As speed increases, a continual upward trend in vibration is evident, with an increasing gradient. To model the potential influence of vehicle speed upon health risk, the vehicle range (in km) before the cumulative A(8) exposure to the patient exceeded a threshold of $0.5m/s^{2}$ was calculated for each vehicle speed. Range at the median exposure level is plotted as the blue dashed trace (on right subplot), and the quartile as the green dashed trace.

From the figure it can be seen that a vehicle speed below 60 km/h would increase A(8) exposure, due to the increased journey time. Optimal vehicle speed is unclear; the median range peaks at a speed of 110 km/h, so it appears that vehicle speed above this results in higher A(8) for a given journey distance, but there is a considerable amount of oscillation in the median line. However, vehicle speed in the 80–110 km/h range appears to be near optimal.

In summary, simply restricting maximum vehicle speed to the UK motorway limit of 113 km/h, and otherwise driving at the highest speed road conditions allow, is likely to result in minimisation of vibration exposure to patients during transport. Transport distances should be limited to below 200 km (125 miles) or shorter if there is likely to be significant travel at <60 km/h (37 mile/h). Routes should be chosen to minimise duration rather than distance.

## Conclusion

We have demonstrated that a custom-made datalogging system for use with MEMS sensors can be successfully deployed in the field to measure shock and vibration during routine neonatal patient transports between UK hospital sites. Data have been recorded at an appropriate sample rate for the duration of a total of 35 journeys, of which 12 were neonatal transports, and the remainder were inter-site vehicle transfers using a manikin together with three different mattress configurations.

Throughout this study, vibration assessment standards designed for adults in the workplace environment have been employed. This was done out of necessity, since commonly agreed methods for assessment of vibration in neonatal patients do not exist. Unfortunately it is not possible to accurately extrapolate the standard metrics to significantly lower body masses, and so the precise levels of hazard identified here are unclear. Standardised assessments for neonatal vibration hazard would aid risk quantification enormously.

Data analysis using $\mathrm{HI}C_{15}$ to quantify linear head acceleration hazard, and ISO 2631-1:1997 for vibration hazard, indicates that shock exposure is negligible but that vibration exposure approaches and sometimes exceeds the $0.5m/s^{2}$ A(8) ‘action value’ threshold for adults in the workplace, as defined in EU regulations. These findings broadly agree with previous studies of vibration during neonatal transport, for example, Campbell et al.^12^ found RMS accelerations between 0.4 and $5.6m/s^{2}$, with the vehicle transports (air transport was also studied) being towards the lower end of this range. Shenai et al.^11^ found values between 2 and $6m/s^{2}$, higher than in this work. The age (1974 model year) of the vehicle used by Shenai suggests improvements in vehicle construction may explain this.

We have not explored influences of vehicle suspension upon vibration conditions, however, the spectral analysis indicates that engine as opposed to wheel vibration is the dominant hazard source, implying that engine mounts are a determining factor as opposed to vehicle suspension.

Comparison of the incubator chassis to forehead gain functions between manikin and neonatal study groups shows good agreement, with <2 dB deviation in the 10–20 Hz band where the majority of vibration hazard is concentrated. This implies that the manikin is an acceptable mechanical model. Manikin data indicate that vibration exposure during incubator transport is not reduced significantly by replacement of the current sponge mattresses with air or gel mattresses. Replacement with air mattresses results in increased vibration exposure under some driving conditions, and the high density of a gel mattress may increase injury risk in a road traffic accident.

Using a GPS datalogger in the vehicle cab, the influence of vehicle speed and road type upon vibration exposure has been investigated. Optimal speed range for minimisation of vibration exposure appears to be between 80 and 110 km/h (50–68 mile/h), with vehicle speed below 60 km/h (37 mile/h) increasing overall exposure due to the longer journey time.

Road type appears to weakly influence vibration, as lower class roads (UK ‘B’ and below) give higher WBV for a given vehicle speed. A reduction in total vibration exposure might be achieved through optimisation of transport routes, for example, longer routes involving ‘A’ and motorway class roads could be adopted in place of shorter routes involving minor roads, although this concept would require further analysis to assess its feasibility.

As the most hazardous vibration is in the 5–20 Hz frequency band, which can be damped with simple mechanical shock absorbers, improved transport trolley and incubator design may be the most promising route for reducing patient’s vibration exposure. The vertical axis resonant frequency of the incubator/mattress system was approximately 10 Hz with a sponge or gel mattresses, with around 5 dB peak gain between incubator chassis and the torso and forehead sites. This frequency lies close to the 14 Hz chassis vibration peak, believed to originate from the engine and transmission, and so is easily excited. Mattress-induced resonance was highlighted in a previous study by Gajendragadkar et al.,^13^ who found vibration gain factors which are comparable to those measured in this study. However, this is the first study to demonstrate, in real patients, that the neonatal head is exposed to mechanical vibration hazard in excess of that simply observed at the incubator chassis.

Changes to the mattress, harness, and incubator system to reduce its resonant frequency and increase damping could potentially decrease RMS WBV by reducing the following: the hazard weighting at resonance (i.e. $W_{d}$ and $W_{k}$), the degree of resonant driving by vehicle chassis vibration, and the gain at resonance, respectively. The air mattress appears promising in this regard; although the air mattress did not significantly decrease WBV, the resonant frequency was reduced to 7 Hz, with lower gain at the 14 Hz chassis vibration peak. A more compliant mattress system with a resonant peak below 7 Hz may result in an overall decrease in vibration exposure, a hypothesis that could be tested experimentally with an underinflated air mattress.

Current transport trolleys use a stiff locking mechanism to clamp directly to the ambulance floor. Although this results in a significant safety improvement in the event of a vehicle accident, vehicle chassis vibration can be transferred directly to the incubator. Hypothetically, an improved coupling incorporating vibration-dampening components could dramatically reduce vibration exposure. As the high exposures found in this and several prior studies are potentially an important mechanism of stress and subsequent brain injury associated with inter-hospital transport in babies, it is recommended that development of such designs is pursued as a priority.
